# Safety and immunogenicity of a booster dose of S-268019-b: Interim findings of a Phase 3, open-label clinical study in Japan

**DOI:** 10.1016/j.jvacx.2023.100390

**Published:** 2023-09-18

**Authors:** Takuhiro Sonoyama, Akari Kamitani, Risa Y. Shibata, Naomi M. Seki, Shinya Omoto, Kenji Igarashi, Mari Ariyasu

**Affiliations:** a Shionogi & Co., Ltd., Drug Development and Regulatory Science Division, 8F, Nissay Yodoyabashi East Bldg., 3-3-13, Imabashi, Chuo-ku, Osaka 541-0042, Japan; b Shionogi & Co., Ltd., Biopharmaceutical Research Division, 1-1, Futaba-cho 3-chome, Toyonaka, Osaka 561-0825, Japan

**Keywords:** Booster vaccine, Cellular immunity, Clinical trial, COVID-19 vaccine, Immunogenicity, Reactogenicity, Safety, Recombinant protein

## Abstract

Despite the initial success of severe acute respiratory syndrome coronavirus 2 (SARS-CoV-2) vaccines in prevention of symptomatic and severe diseases, booster vaccination has become increasingly important with the advent of variants with immune-escaping capacity. Herein, we report the safety and immunogenicity of S-268019-b, comprising SARS-CoV-2 spike protein and a squalene-based adjuvant, as a booster dose. We performed an interim analysis of an open-label, Phase 3 study data until Day 29 following S-268019-b booster in Japanese adults (aged 20–64 years) who had completed primary vaccination with mRNA-1273 and in Japanese elderly (aged ≥ 65 years) who had completed primary vaccination with mRNA-1273 or BNT162b2. Reactogenicity was mild in most participants; no serious treatment-related adverse events were noted. S-268019-b enhanced SARS-CoV-2 neutralizing antibodies, immunoglobulin G antibodies, and predominant T-helper 1-mediated immune reaction in all cohorts, regardless of age, in Japanese participants with prior vaccination with mRNA vaccines.

## Highlights

• S-268019-b is a protein-based subunit vaccine with a squalene-based adjuvant.
• Japanese adults and elderly with prior mRNA vaccination received S-268019-b booster.
• S-268019-b booster dose was well tolerated and enhanced immunity against SARS-CoV-2.
• It also elicited neutralizing antibodies against Omicron and T-cell responses.

## Introduction

Severe acute respiratory syndrome coronavirus 2 (SARS-CoV-2) infection and the ensuing coronavirus disease 2019 (COVID-19) resulted in 625 million confirmed cases and 6.56 million deaths worldwide as of October 2022, with a consistent rise in the cumulative number of infections [1]. Nearly two-thirds of the world’s population is fully vaccinated [1]. However, vaccine-induced neutralizing antibodies wane over time [2], reducing the effectiveness of the primary vaccination against SARS-CoV-2 infection. Preventing severe COVID-19 infection is important, particularly in elderly people and in those with risk factors for morbidity and mortality [3], with the emerging virus variants. Immune response after primary vaccination against Omicron is much lower than that against the ancestral viruses. Booster application enhanced immune response against Omicron compared with the primary vaccination [4]. Evidence suggests that booster vaccination is effective in preventing Omicron-induced symptomatic COVID-19 disease and hospitalization [5].

S-268019-b is a recombinant protein vaccine comprising the S–910823 antigen, a modified recombinant spike protein of SARS-CoV-2 produced in insect cells using the baculovirus expression system, with a squalene-based adjuvant in an oil-in-water emulsion formulation. Safety and immunogenicity of S-268019-b administered as primary vaccination in Japanese participants was successfully demonstrated in a randomized, placebo-controlled Phase 1/2 study [6]. Another randomized Phase 2/3 study showed that S-268019-b was non-inferior to BNT162b2, an approved mRNA vaccine, in producing neutralizing antibodies upon booster application in Japanese adults after the 2-dose BNT162b2 regimen [7].

The majority of the global population, including of Japan, has received SARS-CoV-2 primary vaccination, including mRNA vaccines such as mRNA-1273 and BNT162b2. Considering the importance of data on booster application in a heterologous vaccination schedule, we assessed the safety and immunogenicity of a booster dose of S-268019-b in Japanese adults (aged 20–64 years) who had completed primary vaccination with mRNA-1273 vaccine and in Japanese elderly (aged ≥ 65 years) who had completed primary vaccination with mRNA-1273 or BNT162b2 vaccine. Herein, we report the interim findings of the open-label Phase 3 study evaluating the safety and immunogenicity Day 29 after the booster dose of S-268019-b (data cut-off date for interim findings) in Japanese participants.

## Methods

### Study design and participants

This study was a Phase 3, single-center, non-controlled, open-label study (jRCT2031210613). The study enrolled Japanese participants who had been vaccinated with either mRNA-1273 or BNT162b2 as a primary vaccine 6–8 months before the study, with no documented history of SARS-CoV-2 infection between February 28, 2022 to March 10, 2022. Detailed inclusion and exclusion criteria are described below:

## Inclusion criteria

• Male and female adults (aged between 20 and 64 years) and elderly (aged ≥ 65 years) at the time of signing the informed consent form.
• ≥6 months and ≤ 8 months since the second dose of an approved SARS-CoV-2 vaccine (adults: mRNA-1273 only, elderly: mRNA-1273 or BNT-162b2).

### Exclusion criteria

• Positive test for SARS-CoV-2 infection (as determined by a SARS-CoV-2 antigen test) at screening.
• A history of SARS-CoV-2 infection determined in the interview conducted before the study intervention.
• A contraindication to intramuscular injections or blood draws.
• Previous approved or investigational SARS-CoV-2 vaccination other than 2 doses of approved SARS-CoV-2 vaccines (adults: mRNA-1273 only, elderly: mRNA-1273 or BNT-162b2).
• Use of anti-SARS-CoV-2 monoclonal antibody, immunoglobulin preparations, blood products, or a blood transfusion within 3 months before the study intervention.

**Fig. 1:**
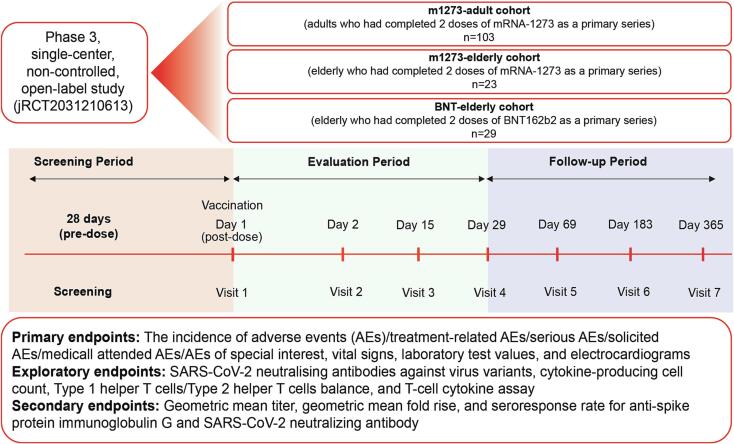
Study design AE, adverse event; SARS-CoV-2, severe acute respiratory syndrome coronavirus 2.

The study comprised 3 cohorts: (1) the m1273-adult cohort included adult participants (aged 20–64 years) who completed 2 doses of mRNA-1273 vaccine as a primary series; (2) the m1273-elderly cohort included elderly participants (aged ≥ 65 years) who completed 2 doses of mRNA-1273 vaccine as a primary series; and (3) the BNT-elderly cohort included elderly participants (aged ≥ 65 years) who completed 2 doses of BNT162b2 vaccine as a primary series (Fig. 1).

### Vaccine dose and administration

S-268019-b injectable contained 40 μg/mL of recombinant SARS-CoV-2 spike protein as an antigen and adjuvanted squalene-based oil-in-water emulsion for injection mixed in a 1:1 ratio (0.25 mL each), resulting in 10 μg/dose. Participants received a 0.5 mL intramuscular injection of S-268019-b on Day 1 and will be monitored for approximately 12 months. The recombinant protein is a full-length SARS-CoV-2 spike protein based on amino acid sequence of the Wuhan-Hu-1 isolate (Genbank MN908947) and has mutations in the furin cleavage site to inhibit S-protein cleavage into S1 and S2 subunits, as well as two substituted proline residues to improve prefusion conformation stability of the S-protein trimers [8].

### Compliance

The study was conducted in accordance with the protocol, the Declaration of Helsinki and Council for International Organizations of Medical Sciences (CIOMS) International Ethical Guidelines, the International Council for Harmonisation of Technical Requirements for Pharmaceuticals for Human Use (ICH) Good Clinical Practice Guidelines, and other applicable laws and regulations. The study was approved by Institutional Review Board. All participants provided the written informed consent.

### Safety information collection

All adverse events (AEs)/serious AEs (SAEs) were collected from the date of signing the ICF until the end-of-study/early discontinuation examination. After the initial AE/SAE report, the investigator was required to proactively follow each participant at subsequent visits/contacts.

The investigator referred to the participant diary for assessment of solicited AEs developed within 7 days after study intervention (Day 1 to Day 8). The following AEs were collected as solicited AEs (solicited systemic AEs and solicited local AEs) within 7 days after study intervention in this study.

Solicited systemic adverse events

–Fever

–Nausea/vomiting

–Diarrhea

–Headache

–Fatigue

–Myalgia

–Arthralgia

–Chills

Solicited local adverse events

–Pain

–Erythema/redness

–Induration

–Swelling

Participants recorded their body temperature and the status of symptoms falling under the category of solicited AEs in the participant diary every day at the same time as possible within 7 days after the study intervention. The intensity of solicited systemic AEs and pain was assessed, and the longest diameter was measured for erythema/redness, induration, and swelling, and recorded in the participant diary. The investigator assessed AEs in reference to the participant diary and medical judgment based on physical examination and interview to assess their severity and causal relationship. However, if the participant died due to a solicited AE, this event was considered of Grade 5 severity.

Medically attended AE (MAAE)

MAAE was defined as an AE that resulted in a visit to/from a health care professional (eg, hospital, emergency room, or home) because of the AE. MAAEs were recorded and reported.

AE of special interest (AESI)

Potential immune-mediated diseases were collected as AESIs of S-268019. Serious AESIs were recorded and reported. The potential AESIs are described in Supplementary Table 1.

Methodological details for cytopathic effect-based virus neutralization test, neutralization assay against live virus and pseudotyped virus variants, intracellular cytokine staining by flow cytometry, and anti-spike protein immunoglobulin G (IgG) titer measurement assays have been described previously by Shinkai et al [7].

### Cytopathic effect-based virus neutralization test

Neutralizing antibody levels were assessed with the SARS-CoV-2 JPN/TY/WK-521 ancestral wildtype strain (kindly provided by National Institute of Infectious Diseases, Japan) and transmembrane serine protease-2 (TMPRSS-2)-expressing VeroE6 cells. Heat-inactivated vaccinated sera and convalescent plasma (56 °C for approximately 30 min to remove non-specific inhibitors) were 2-fold serially diluted. Each sera/plasma sample was then mixed with 1:1 live virus suspension containing 100 times the median tissue culture infectious dose (TCID_50_)/well, and incubated for 1 h at 37 °C. The mixture of sample and virus was dispensed into each well of 96-well culture plates, and VeroE6/TMPRSS2 cell suspension (1 × 10^4^ cells/well) was added to the wells. The plates were incubated at 37 °C for 5 days with 5% CO_2_, followed by examination for cytopathic effect under a microscope. Virus neutralization titer was defined as the reciprocal of the highest dilution resulting in equal to or more than 50% cell viability.

### Neutralization assay against live virus variants

Neutralizing antibody levels were assessed with the SARS-CoV-2 wildtype strain (WK-521), Delta strain (TY11-927-P1), and Omicron strain (TY38-873), kindly provided by the National Institute of Infectious Diseases, Japan, using the VeroE6/TMPRSS-2 cells. Heat-inactivated vaccinated sera and convalescent plasma (56 °C for approximately 30 min to remove non-specific inhibitors) were 2-fold serially diluted. Each sera/plasma sample was then mixed with 1:1 live virus suspension containing either 2000x, 2000x, or 200,000x TCID_50_/well for wildtype, Delta, and Omicron strain, respectively, and incubated for 1 h at room temperature for neutralization. The mixture of sample and virus was dispensed (100 µL) into each well of culture plates in duplicates, and 100 µL of VeroE6/TMPRSS2 cell suspension (3 × 10^4^ cells/well) was added to the wells. The plates were incubated at 37 °C for 3 days with 5% CO_2_, followed by examination for cytopathic effect using CellTiter-Glo® 2.0 reagent (G9243, Promega) as per manufacturer’s instructions. Virus neutralization titer was defined as the reciprocal of the highest dilution resulting in equal to or more than 50% cell viability.

### Pseudotyped virus neutralization assay

The stocks of D614G, Delta, and Omicron pseudotyped lentiviruses were diluted with the assay medium to prepare the viral suspensions of the same viral RNA copies per milliliter. Eleven serial 2-fold dilutions of heat-inactivated sera or control plasma were mixed with an equal volume of viral suspension, followed by incubating for approximately 1 h at 37 °C for neutralization. After incubation, the mixture of sample and virus were added in duplicate to HEK293T stably expressing human ACE2 and TMPRSS2 cells, which were seeded into 96-well plates the day before the neutralization assay. Only virus suspension was added to Virus Control (VC) wells, which were placed on each plate. After incubating the plates at 37 °C with 5% CO_2_ for 2 days, cells were lysed and subjected to luciferase assay to measure Luciferase gene expression caused by lentiviral transduction. The intensity of luminescence was measured by a microplate reader. Percent neutralization was calculated as the difference between relative light units (RLUs) of VC wells and test sample wells:

%Neutralization=100%×[1-(meanRLUofduplicatesamplewellsÃ·meanRLUofVCwells)]

The dilution factor achieving 50% of neutralization (50% neutralization titer; NT_50_) was calculated by using the XLfit 5.3.1.3 software. When the percentage neutralization was less than 50% at the first dilution, the NT_50_ was expressed as the half of the first dilution factor. Geometric mean of the NT_50_ for each pseudovirus strain was calculated.

### Anti-spike protein IgG titer measurement

Enzyme-linked immunosorbent assay (ELISA) was used to measure anti-spike protein IgG titers using duplicated samples. Full-length trimeric SARS-CoV-2 spike protein (BioServUK Ltd, BSV-COV-PR-35) was used as the immobilized antigen, and horseradish peroxidase conjugated goat-anti-human IgG (H + L) antibody (Invitrogen, A18811) was used as the detection antibody. Sample absorbance was measured as the difference between absorbance values at 405 nm and 490 nm, and the mean and coefficient of variation (CV) of absorbance for each duplicate were determined. The highest dilution factor with mean absorbance value more than or equal to the cutoff absorbance was considered the antibody titer for that sample.

### Intracellular cytokine staining (ICS) by flow cytometry

Cytokine-producing T cells were identified by intracellular cytokine staining. Human peripheral blood mononuclear cells (PBMCs), thawed and rested for 4–5 h in R10 supplemented medium, were restimulated (1.0 × 10^6^ cells per well) with overlapping peptide pools of SARS-CoV-2 S (Miltenyi Biotec) and epitope peptide pools (Shionogi & Co., Ltd.) in the presence of Protein Transport Inhibitor Cocktail (Thermo Fisher Scientific K.K.) for 16 h at 37 °C. Controls were treated with a dimethyl sulfoxide-containing medium. Cells were stained for viability and surface markers (CD3 BV421 [BioLegend]; CD4 BV510 [BioLegend]; CD8 BB515 [BD Biosciences]) in staining buffer and Brilliant Stain Buffer Plus (BSB Plus, BD Horizon, according to the manufacturer’s instructions) for 16–20 h in a refrigerator. Next, the samples were fixed and permeabilized using the Cytofix/Cytoperm kit, according to the manufacturer’s instructions (BD Biosciences). Intracellular staining for interferon gamma (IFN-γ) and interleukin (IL) (IFN-γ PE–Cy7 [BD Biosciences], IL-2 BB700 [BD Biosciences], IL-4 APC [BioLegend], and IL-5 PE [BioLegend]) was performed in the Perm/Wash buffer supplemented with BSB Plus (BD Horizon, according to the manufacturer’s instructions) for 16–20 h in a refrigerator. Samples were acquired on BD FACSCantoTM II (BD Biosciences) and analyzed with the FlowJo software version 7.6.5 (Becton, Dickinson and Company).

### Outcomes

Primary endpoints included the incidence of AEs/TRAEs/SAEs/solicited AEs/MAAEs/ AESIs, vital signs, laboratory test values, and electrocardiograms. Secondary endpoints were related to immunogenicity, including geometric mean titer (GMT), geometric mean fold rise (GMFR), and seroresponse rate (proportion of participants with a ≥ 4-fold rise from baseline in SARS-CoV-2 neutralizing antibody titer and anti-spike protein immunoglobulin G [IgG] antibodies). Exploratory endpoints included immunological indices such as cytokine-producing cell count, T-cell cytokine assay to assess T-helper cell 1 (Th1)/Th2 balance, and neutralizing antibody titer against pseudovirus variants (B.1 [D614G], Delta, BA.1, BA.2, BA.2.12.1, and BA.4/5) and live viruses (BA.1, BA.4, and BA.5).

Safety analyses were conducted with the safety analysis set which included participants who received the vaccine. Immunogenicity was analyzed in the immunogenicity subset which included participants who received the vaccine and met all of the following criteria: availability of ≥ 1 post-vaccination immunogenicity data, completion of the second vaccination with an approved SARS-CoV-2 vaccine (adults: mRNA-1273 only, elderly: BNT162b2 or mRNA-1273) at screening, and a negative anti-SARS-CoV-2 N-protein antibody test result at screening.

### Sample size determination

The target number of adults aged between 20 and 64 years was 100 to evaluate at the comparable accuracy to booster dose study of S-268019 [7], and the target number of older adults aged at least 65 years was 25 for each cohort in consideration of the feasibility. Probability of occurrence of adverse events with an incidence of 2% in at least 1 participant was 95% among the safety analysis set of 150 participants.

### Statistical analyses

All analyses were descriptive; no confirmatory hypothesis testing was performed. The GMT or GMFR and the corresponding 95% CI were calculated by back transformation of the arithmetic mean and 95% CI of the log-transformed titers or the change from baseline in log-transformed titers, respectively. The 95% CIs for seroresponse rates were calculated using the Clopper-Pearson method.

## Results

### Participant disposition

**Fig. 2:**
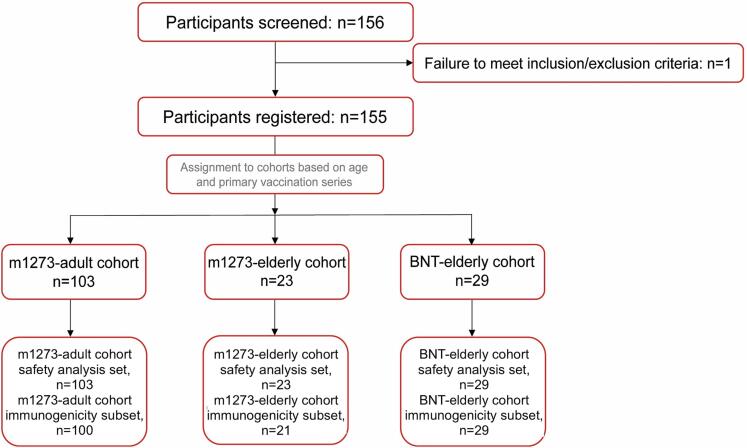
Participant disposition The safety analysis set included participants who received the vaccine. The immunogenicity subset included participants who received the vaccine and met all of the following criteria: availability of ≥ 1 post-vaccination immunogenicity data, completion of the second vaccination with an approved SARS-CoV-2 vaccine (adults: mRNA-1273 only, older adults: BNT162b2 or mRNA-1273) at screening, and a negative anti-SARS-CoV-2 N-protein antibody test result at screening. All 5 participants who had a positive antibody test for SARS-CoV-2 were excluded from safety analysis set for the immunogenicity subset.

The m1273-adult, m1273-elderly, and BNT-elderly cohorts included 103 (49.5% male), 23 (65.2% male), and 29 (75.9% male) participants, respectively (Fig. 2**;** Table 1). All participants were Asian and had received anti-SARS-CoV-2 vaccination within ≥ 180 to less than 240 days after the second vaccine. While safety analysis included all participants who received the vaccine; notably, the immunogenicity subset included participants who received the vaccine and met all of the following criteria: availability of ≥ 1 post-vaccination immunogenicity data, completion of the second vaccination with an approved SARS-CoV-2 vaccine (adults: mRNA-1273 only, older adults: BNT162b2 or mRNA-1273) at screening, and a negative anti-SARS-CoV-2 N-protein antibody test result at screening. Therefore 5 participants with positivity by antibody test for SARS-CoV-2 at screening were excluded from the immunogenicity subset.

**Table 1:** Demographics and baseline characteristics (safety analysis population).

	**m1273-adult cohort** **(n = 103)**	**m1273-elderly cohort** **(n = 23)**	**BNT-elderly cohort** **(n = 29)**
**Age (years), mean (SD)**	45.7 (10.9)	68.4 (3.8)	68.4 (3.2)
<30	9 (8.7)	0 (0)	0 (0)
≥30 to < 40	17 (16.5)	0 (0)	0 (0)
≥40 to < 50	32 (31.1)	0 (0)	0 (0)
≥50 to < 60	36 (35.0)	0 (0)	0 (0)
≥60 to < 65	9 (8.7)	0 (0)	0 (0)
≥65	0 (0)	23 (100.0)	29 (100.0)
**Height (cm), mean (SD)**	165.4 (8.2)	163.5 (8.2)	164.9 (8.2)
**Weight (kg), mean (SD)**	63.5 (12.9)	68.3 (12.4)	63.2 (13.0)
**BMI (kg/m^2^), mean (SD)**	23.0 (3.5)	25.4 (3.2)	23.1 (3.9)
**Sex**			
Male	51 (49.5)	15 (65.2)	22 (75.9)
Female	52 (50.5)	8 (34.8)	7 (24.1)
**Ethnicity**			
Not Hispanic or Latino	103 (100.0)	23 (100.0)	29 (100.0)
**Race**			
Asian	103 (100.0)	23 (100.0)	29 (100.0)
**No previous SARS-CoV-2 infection**	103 (100.0)	23 (100.0)	29 (100.0)
**Previous vaccination against SARS-CoV-2**	103 (100.0)	23 (100.0)	29 (100.0)
**Time from the second dose of SARS-CoV-2 vaccine as a primary series (days)**			
<180	0 (0)	0 (0)	0 (0)
≥180 to < 210	62 (60.2)	0 (0)	6 (20.7)
≥210 to < 240	41 (39.8)	18 (78.3)	18 (62.1)
≥240	0 (0)	5 (21.7)	5 (17.2)
**Current/former smokers**	12 (11.7)	0 (0)	3 (10.3)

BMI, body mass index; COVID-19, Coronavirus disease 2019; SARS-CoV-2, severe acute respiratory syndrome coronavirus 2.

Data are reported as n (%) unless stated otherwise.

The m1273-adult cohort comprised adults (20–64 years) who had completed 2 doses of mRNA-1273 as a primary series.

The m1273-elderly cohort comprised elderly (≥65 years) who had completed 2 doses of mRNA-1273 as a primary series.

The BNT-elderly comprised elderly (≥65 years) who had completed 2 doses of BNT162b2 as a primary series.

### Safety

S-268019-b was well-tolerated as a booster dose in all cohorts. The incidence of AEs was 98.1%, 87.0%, and 79.3% in the m1273-adult, m1273-elderly, and BNT-elderly cohorts, respectively. Most were treatment-related (Table 2). No mortality and AESIs were reported by the data cutoff date and just 1 non-treatment-related SAE of Grade 3 pancreatic carcinoma was reported in 1 participant (4.3%) in the m1273-elderly cohort. The incidence of solicited systemic TRAEs was 68.0%, 56.5%, and 34.5% in the m1273-adult, m1273-elderly, and BNT-elderly cohorts, respectively (Table 3). No Grade 3 or above solicited systemic AEs was reported in any cohort. The incidence of solicited local TRAEs was 94.2%, 82.6%, and 79.3% in the m1273-adult, m1273-elderly, and BNT-elderly cohorts, respectively. Grade 3 solicited local AEs reported included swelling (n = 2), erythema/redness (n = 1), and vaccination site pain (n = 1) in the m1273-adult cohort, and erythema (n = 1) in the m1273-elderly cohort. Median time to onset of treatment-related systemic AEs was 2 days in all cohorts, but 1 day in the m1273-adult cohort and 2 days in the m1273-elderly and BNT-elderly cohorts for treatment-related solicited local AEs. All TRAEs were resolved by the data cutoff date.

**Table 2:** Incidence of treatment-emergent adverse events.

	**m1273-adult cohort** **(n = 103)**	**m1273-elderly cohort** **(n = 23)**	**BNT-elderly cohort** **(n = 29)**
TEAEs	101 (98.1)	20 (87.0)	23 (79.3)
Death	0 (0)	0 (0)	0 (0)
Other serious AEs	0 (0)	1 (4.3)	0 (0)
AEs of special interest	0 (0)	0 (0)	0 (0)
Medically attended AEs	5 (4.9)	1 (4.3)	0 (0)
Unsolicited AEs	14 (13.6)	3 (13.0)	2 (6.9)
TRAEs	100 (97.1)	20 (87.0)	23 (79.3)
Death	0 (0)	0 (0)	0 (0)
Other serious AEs	0 (0)	0 (0)	0 (0)
AEs of special interest	0 (0)	0 (0)	0 (0)
Medically attended AEs	3 (2.9)	0 (0)	0 (0)
Unsolicited AEs	5 (4.9)	0 (0)	0 (0)

AE, adverse event; TEAE, treatment-emergent AE; TRAE, treatment-related AE.

Data are presented as n (%).

The m1273-adult cohort comprised adults (20–64 years) who had completed 2 doses of mRNA-1273 as a primary series.

The m1273-elderly cohort comprised elderly (≥65 years) who had completed 2 doses of mRNA-1273 as a primary series.

The BNT-elderly comprised elderly (≥65 years) who had completed 2 doses of BNT162b2 as a primary series.

**Table 3:** Solicited systemic and local TRAEs by severity.

	**m1273-adult cohort (n = 103)**				**m1273-elderly cohort (n = 23)**				**BNT-elderly cohort (n = 29)**			
	**Any grade**	**Grade 1**	**Grade 2**	**Grade 3**	**Any grade**	**Grade 1**	**Grade 2**	**Grade 3**	**Any grade**	**Grade 1**	**Grade 2**	**Grade 3**
Any systemic solicited TRAEs	70 (68.0)	58 (56.3)	12 (11.7)	0 (0)	13 (56.5)	12 (52.2)	12 (11.7)	0 (0)	10 (34.5)	9 (31.0)	1 (3.4)	0 (0)
Fever	21 (20.4)	17 (16.5)	4 (3.9)	0 (0)	1 (4.3)	1 (4.3)	0 (0)	0 (0)	0 (0)	0 (0)	0 (0)	0 (0)
Nausea/vomiting	21 (20.4)	20 (19.4)	1 (1.0)	0 (0)	4 (17.4)	4 (17.4)	0 (0)	0 (0)	2 (6.9)	2 (6.9)	0 (0)	0 (0)
Diarrhea	10 (9.7)	10 (9.7)	0 (0)	0 (0)	2 (8.7)	1 (4.3)	1 (4.3)	0 (0)	1 (3.4)	0 (0)	1 (3.4)	0 (0)
Headache	34 (33.0)	30 (29.1)	4 (3.9)	0 (0)	4 (17.4)	4 (17.4)	0 (0)	0 (0)	7 (24.1)	7 (24.1)	0 (0)	0 (0)
Fatigue	51 (49.5)	47 (45.6)	4 (3.9)	0 (0)	7 (30.4)	6 (26.1)	1 (4.3)	0 (0)	5 (17.2)	5 (17.2)	0 (0)	0 (0)
Myalgia	9 (8.7)	9 (8.7)	0 (0)	0 (0)	2 (8.7)	2 (8.7)	0 (0)	0 (0)	1 (3.4)	1 (3.4)	0 (0)	0 (0)
Arthralgia	19 (18.4)	18 (17.5)	1 (1.0)	0 (0)	2 (8.7)	2 (8.7)	0 (0)	0 (0)	1 (3.4)	1 (3.4)	0 (0)	0 (0)
Chills	23 (22.3)	22 (21.4)	1 (1.0)	0 (0)	2 (8.7)	2 (8.7)	0 (0)	0 (0)	4 (13.8)	4 (13.8)	0 (0)	0 (0)
Any local solicited TRAEs	97 (94.2)	75 (72.8)	19 (18.4)	3 (2.9)	19 (82.6)	18 (78.3)	0 (0)	1 (4.3)	23 (79.3)	18 (62.1)	5 (17.2)	0 (0)
Pain	95 (92.2)	87 (84.5)	7 (6.8)	1 (1.0)	17 (73.9)	17 (73.9)	0 (0)	0 (0)	22 (75.9)	21 (72.4)	1 (3.4)	0 (0)
Erythema/redness	46 (44.7)	37 (35.9)	8 (7.8)	1 (1.0)	10 (43.5)	9 (39.1)	0 (0)	1 (4.3)	11 (37.9)	9 (31.0	2 (6.9)	0 (0)
Induration	31 (30.1)	20 (19.4)	11 (10.7)	0 (0)	8 (34.8)	8 (34.8)	0 (0)	0 (0)	10 (34.5)	8 (27.6)	2 (6.9)	0 (0)
Swelling	32 (31.1)	17 (16.5)	13 (12.6)	2 (1.9)	4 (17.4)	4 (17.4)	0 (0)	0 (0)	8 (27.6)	6 (20.7)	2 (6.9)	0 (0)

AE, adverse event; TRAE, treatment-related adverse event.

Data are presented at n (%) unless stated otherwise.

Solicited AEs were collected within 7 days after study intervention (Day 1 to Day 8 in the study).

For summarization of “Participants with any treatment-related solicited local AEs,” in case that a participant experiences more than 1 treatment-related solicited local AE, the participant was counted only once for a category of severity when experiencing treatment-related solicited local AEs falling within the same category, and the participant was counted only once for the severest category in the order of severe manifestation, 'Grade 5', 'Grade 4', 'Grade 3', 'Grade 2' and 'Grade 1' when experiencing treatment-related solicited local AEs in different categories of severity.

The m1273-adult cohort comprised adults (20–64 years) who had completed 2 doses of mRNA-1273 as a primary series.

The m1273-elderly cohort comprised elderly (≥65 years) who had completed 2 doses of mRNA-1273 as a primary series.

The BNT-elderly comprised elderly (≥65 years) who had completed 2 doses of BNT162b2 as a primary series.

### Immunogenicity

**Fig. 3:**
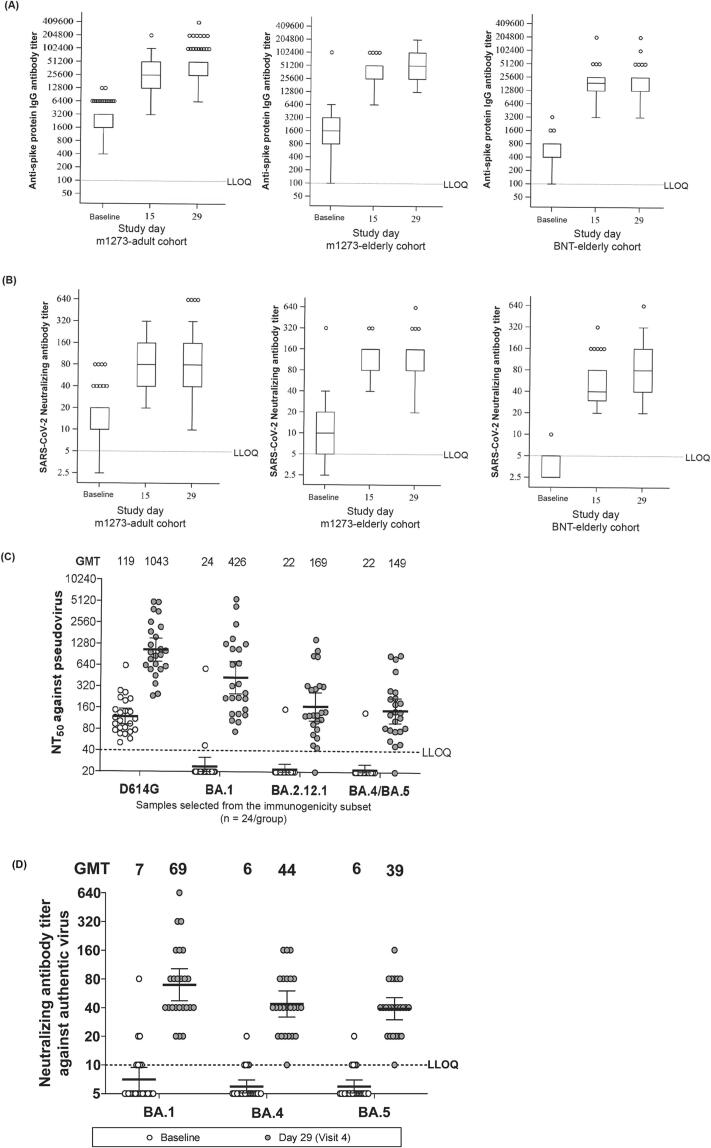
Cohort-wise GMT for (A) anti-S protein IgG antibody titer, (B) neutralization antibody titer, (C) NT_50_ against pseudotype virus variants, and (D) neutralization antibody titer against live virus GMT, geometric mean titer; IgG, immunoglobulin G; IQR, interquartile range; LLOQ, lower limit of quantification; NT_50_, 50% neutralization titer; SARS-CoV-2, severe acute respiratory syndrome coronavirus 2. Titer values reported as below the LLOQ are replaced by 0.5 × LLOQ. The 95% CI were constructed using Student’s t distribution for log-transformed titers The m1273-adult cohort comprised adults (20–64 years) who had completed 2 doses of mRNA-1273 as a primary series. The m1273-elderly cohort comprised elderly (≥65 years) who had completed 2 doses of mRNA-1273 as a primary series The BNT-elderly cohort comprised elderly (≥65 years) who had completed 2 doses of BNT162b2 as a primary series. The white and grey circles in Panel C represent individual values for the baseline and Day 29 (Visit 4), respectively. In panel C, the samples selected from the immunogenicity subset (n = 24/group) were assessed. The sampling ensured no significant differences existed in age and neutralizing antibody titer against live wild-type virus on Day 29 compared with the entire cohort. Box: The bottom and top edges of the box are located at the 25th and 75th percentiles of the sample. Bar: Minimum observation above lower fence (MIN) and maximum observation below upper fence (MAX). The lower fence (MIN) is 1.5 times below the 25th percentile. The upper fence (MAX) is 1.5 times above the 75th percentile. Dots: Outliers that are observations that are more extreme than the upper and lower fences (±1.5 times IQR). IQR is the range between the 25th and 75th percentiles.

Baseline GMT of SARS-CoV-2 neutralizing antibody was lower in the BNT-elderly cohort (3.41) than in the m1273-adult and m1273-elderly cohorts (12.14 and 10.34, respectively; Fig. 3**,** Table 4). The GMT of SARS-CoV-2 neutralizing antibody in all cohorts increased from baseline to Day 29 post–vaccination. The GMTs (95% CI) on Day 29 were 99.35 (83.44–118.29), 129.96 (88.79–190.22), and 76.14 (55.38–104.67) in the m1273-adult, m1273-elderly, and BNT-elderly cohorts, respectively. The GMFRs (95% CI) on Day 29 were 8.06 (6.54–9.92), 11.71 (7.45–18.41), and 22.07 (16.12–30.23) in the m1273-adult, m1273-elderly, and BNT-elderly cohorts, respectively (Table 5). The seroresponse rates of SARS-CoV-2 neutralizing antibody titer were greater than 80% in all cohorts on Day 15. The high seroresponse rates were maintained through Day 29.

**Table 4:** GMT for anti-S protein IgG antibody titer and neutralization antibody titer.

**Timepoint, Assessment**	**m1273-adult cohort** **(n = 100)**	**m1273-elderly cohort** **(n = 21)**	**BNT-elderly cohort** **(n = 29)**	
**GMT for SARS-CoV-2 neutralizing antibodies**				
**Baseline**				
n	100	21	29	
GMT (95% CI)^a^	12.14 (10.53–14.00)	10.34 (6.13–17.44)	3.41 (2.93–3.97)	
**Day 15**				
n	98	19	28	
GMT (95% CI)^a^	92.16 (79.70–106.56)	115.22 (86.99–152.61)	52.52 (38.39–71.84)	
**Day 29**				
n	96	20	28	
GMT (95% CI)^a^	99.35 (83.44–118.29)	129.96 (88.79–190.22)	76.14 (55.38–104.67)	
**GMT for anti-spike protein IgG antibodies**				
**Baseline**				
n	100	21	29	
GMT (95% CI)^a^	2039.3 (1763.2–2358.6)	1766.5 (937.8–3327.8)	508.0 (389.3–663.0)	
**Day 15**				
n	98	19	28	
GMT (95% CI)^a^	28667.4 (24477.6–33574.2)	38240.2 (26292.3–55617.5)	17227.5 (12183.4–24360.0)	
**Day 29**				
n	96	20	28	
GMT (95% CI)^a^	34171.9 (28792.1–40556.9)	49455.9 (32689.2–74822.5)	21000.6 (14917.8–29563.6)	

GMT, geometric mean titer; IgG, immunoglobulin G; LLOQ, lower limit of quantification; SARS-CoV-2, severe acute respiratory syndrome coronavirus 2.

LLOQ are replaced by 0.5 × LLOQ.

Titer values reported as below the LLOQ are replaced by 0.5 × LLOQ.

The m1273-adult cohort comprised adults (20–64 years) who had completed 2 doses of mRNA-1273 as a primary series.

The m1273-elderly cohort comprised elderly (≥65 years) who had completed 2 doses of mRNA-1273 as a primary series.

The BNT-elderly comprised elderly (≥65 years) who had completed 2 doses of BNT162b2 as a primary series.

a The 95% CI is calculated based on the Student's *t* distribution of the change from baseline in the log-transformed values, then back transformed to the original scale.

**Table 5:** GMFR and seroresponse rates for SARS-CoV-2 neutralizing antibodies and anti-spike protein IgG antibodies (immunogenicity subset).

**Timepoint, Assessment**	**m1273-adult cohort** **(n = 100)**	**m1273-elderly cohort** **(n = 21)**	**BNT-elderly cohort** **(n = 29)**
**GMFR for SARS-CoV-2 neutralizing antibodies**			
**Day 15**			
n	98	19	28
GMFR (95% CI)^a^	7.56 (6.27–9.11)	10.71 (6.26–18.34)	15.23 (11.48–20.20)
**Day 29**			
n	96	20	28
GMFR (95% CI)^a^	8.06 (6.54–9.92)	11.71 (7.45–18.41)	22.07 (16.12–30.23)
**Seroresponse rates**^b^**for SARS-CoV-2 neutralizing antibodies**			
**Day 15**			
n	98	19	28
Seroresponse rate (95% CI)^c^	84.7 (76.0–91.2)	89.5 (66.9–98.7)	100.0 (87.7–100.0)
**Day 29**			
n	96	20	28
Seroresponse rate (95% CI)^c^	86.5 (78.0–92.6)	95.0 (75.1–99.9)	100.0 (87.7–100.0)
**GMFR for anti-spike protein IgG antibodies**			
**Day 15**			
n	98	19	28
GMFR (95% CI)^a^	13.99 (11.53–16.97)	21.42 (11.97–38.34)	32.80 (23.75–45.30)
**Day 29**			
n	96	20	28
GMFR (95% CI)^a^	16.83 (13.71–20.66)	25.99 (15.03–44.96)	39.99 (28.36–56.38)
**Seroresponse rates**^b^**for anti-spike protein IgG antibodies**			
**Day 15**			
n	98	19	28
Seroresponse rate (95% CI)^c^	94.9 (88.5–98.3)	94.7 (74.0–99.9)	100.0 (87.7–100.0)
**Day 29**			
n	96	20	28
Seroresponse rate (95% CI)^c^	97.9 (92.7–99.7)	95.0 (75.1–99.9)	100.0 (87.7–100.0)

GMFR, geometric mean fold rise; IgG, immunoglobulin G; LLOQ, lower limit of quantification; SARS-CoV-2, severe acute respiratory syndrome coronavirus 2.

Titer values reported as below the LLOQ are replaced by 0.5 × LLOQ.

The m1273-adult cohort comprised adults (20–64 years) who had completed 2 doses of mRNA-1273 as a primary series.

The m1273-elderly cohort comprised elderly (≥65 years) who had completed 2 doses of mRNA-1273 as a primary series.

The BNT-elderly comprised elderly (≥65 years) who had completed 2 doses of BNT162b2 as a primary series.

a The 95% CI is calculated based on the Student's *t* distribution of the change from baseline in the log-transformed values, then back transformed to the original scale.

b Seroresponse is defined as a 4-times or higher rise from baseline in anti-spike protein IgG antibody titer or neutralizing antibody titer, where titer values reported as below the LLOQ are replaced by 0.5 × LLOQ.

c The 95% CI is calculated using the Clopper-Pearson method.

Baseline GMT of anti-spike protein IgG was lower in the BNT-elderly cohort (508.0) than in the m1273-adult and m1273-elderly cohorts (2039.3 and 1766.5, respectively; Fig. 3**,** Table 4). The GMTs of anti-spike protein IgG antibody in all cohorts increased from baseline to Day 29 post–vaccination. The GMTs (95% CI) on Day 29 were 34171.9 (28792.1–40556.9), 49455.9 (32689.2–74822.5), and 21000.6 (14917.8–29563.6) in the m1273-adult, m-1273-elderly, and BNT-elderly cohorts, respectively. The GMFRs (95% CI) on Day 29 were 16.83 (13.71–20.66), 25.99 (15.03–44.96), and 39.99 (28.36–56.38) in the m1273-adult, m1273-elderly, and BNT-elderly cohorts, respectively (Table 5). The seroresponse rates of anti-spike protein IgG antibody titer increased greater than 90% in all cohorts on Day 15 and were maintained through Day 29.

**Supplementary figure 1:**
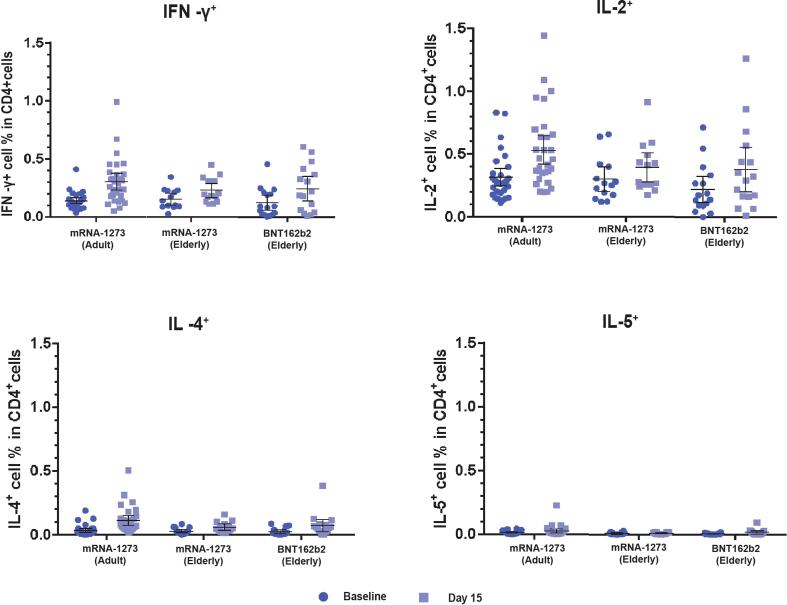
Th1- and Th2-type cytokines by flow cytometry The m1273-adult cohort comprised adults (20-64 years) who had completed 2 doses of mRNA-1273 as a primary series.The m1273-elderly cohort comprised elderly (≥65 years) who had completed 2 doses of mRNA-1273 as a primary series.The BNT-elderly comprised elderly (≥65 years) who had completed 2 doses of BNT162b2 as a primary series.CD, cluster of differentiation cells; IFN- γ, interferon gamma; IL- interleukin.

**Supplementary figure 2:**
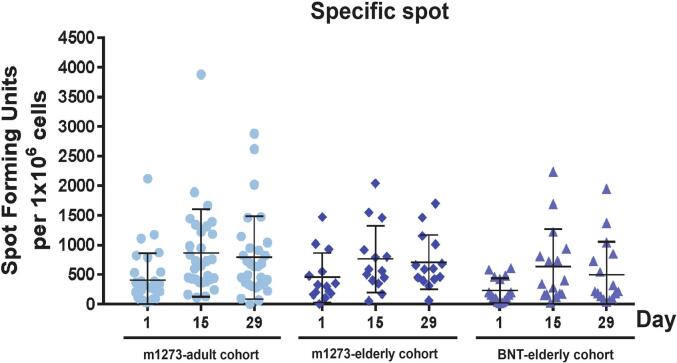
IFN-γ spot-forming units in response to different conditions: ELISPOT assay The m1273-adult cohort comprised adults (20-64 years) who had completed 2 doses of mRNA-1273 as a primary series.The m1273-elderly cohort comprised elderly (≥65 years) who had completed 2 doses of mRNA-1273 as a primary series.The BNT-elderly comprised elderly (≥65 years) who had completed 2 doses of BNT162b2 as a primary series. IFN-γ, interferon gamma

A predominant Th1-mediated immune reaction was observed as reflected in the antigen-specific polyfunctional CD4 + T-cell responses with interferon gamma (IFN-γ) and interleukin 2 (IL-2) production on spike peptide stimulation in all 3 cohorts; Supplementary Fig. 1). IFN-γ levels also increased substantially on Days 15 and 29 in participants from all cohorts in ELISPOT assay **(**Supplementary Fig. 2). The extent of elevation of IL-4 and IL-5 positive cells was lower than that of IFN-gamma and IL-2. (Supplementary Fig. 1).

### Immunogenicity against virus variants

S-268019-b booster elicited neutralizing antibodies against SARS-CoV-2 pseudovirus and live virus variants. At baseline, GMTs of neutralizing antibodies against BA.1, BA.2.12.1, and BA.4/5 were below LLOQ. However, after boosting with S-268019-b, the GMTs increased substantially on Day 29 (Fig. 3). Compared with D614G, the Delta and Omicron pseudovirus variants showed a 1.5- to 7-fold decrease in the reciprocal dilution of sera required to inhibit viral infection by 50% (NT_50_), and the neutralization potency was lower for BA.4/5 than for BA.1. (Fig. 3). For the assay using live viruses, S-268019-b booster was also able to elicit neutralizing antibodies against variants including BA.4 and BA.5 live viruses (Fig. 3**)**.

## Discussion

In this open-label clinical study, booster vaccination with S-268019-b was well tolerated and elicited neutralizing antibodies in Japanese adults and elderly participants who had completed primary BNT162b2 or mRNA-1273 vaccination regimen. The immunogenicity and safety results of the current study support the application of S-268019-b as a booster dose in people with primary vaccination with mRNA vaccines. In this study, we did not enroll adult participants who had completed primary vaccination with BNT162b2, because this population was already assessed in another study [7].

S-268019-b was well tolerated in all cohorts regardless of age and choice of primary vaccination, evident in low incidence of non-solicited TRAEs. No treatment-related SAE or AESI was reported until the cutoff date. Most frequent solicited TRAEs were pain at injection site, fatigue, and headache, which concur with previously published papers on booster applications of S-268019-b and other vaccines [7], [9], [10]. Importantly, most solicited AEs post-vaccination were mild (Grade 1–2) in severity and resolved before the cutoff date. A lower incidence of systemic TRAEs was noted in elderly than in adult participants (34.5% and 56.5% in the m1273-elderly and BNT-elderly cohorts, respectively, vs 68.0% in the m1273-adult cohort). This concurs with results from the S-268019-b Phase 2/3 open-label study (manuscript in preparation) and studies with other vaccines [11], [12]. However, the incidence of local TRAEs was comparable between adult (94.2%) and elderly (82.6%) participants.

Immunogenicity results further support booster dose of S-268019-b in subjects who have completed primary vaccination. S-268019-b was previously shown to be safe and non-inferior to BNT162b2 in terms of neutralizing antibody titer following booster administration among Japanese adult participants who were fully vaccinated with 2 BNT162b2 injections [7]. The m1273-adults cohort showed neutralizing antibody titers comparable with those reported by Shinkai et al. [7]. Baseline GMTs for neutralizing antibodies in adult and elderly participants who completed the second dose of mRNA-1273 as a primary vaccine were generally comparable. On Day 29, GMT was numerically higher in the elderly than in adults. Although neutralizing antibodies are generally higher in adults compared with the elderly in the vaccine-naïve participants as a part of two-vaccine regimen [13], [14], understanding the magnitude of response after a booster application in adults and elderly population needs more studies. Moreover, the small sample size might have affected the results. A large phase 3 study will shed light on the potential immunogenic differences between age groups and the plausible mechanisms behind them.

Importantly, boosting with S-268019-b elicited humoral immune responses against Omicron variants, including sublineages BA.2.12.1 and BA.4/5 which have replaced the older sublineages, BA.1 and BA.2. Our findings are highly relevant in context of a study in which primary vaccination with 2 doses of BNT162b2 or ChAdOx1 nCoV-19 provided limited protection against infection with Omicron [15], whereas evidence suggests that booster application offered substantial protection against symptomatic disease and may offer greater protection against severe and fatal disease [15]. Following booster vaccination of S-268019-b, neutralizing antibody titers against all assessed variants were higher compared with those from baseline, although neutralizing antibody titers against BA.1, BA.2, BA.4, and BA.5 were 1.7–7-fold lower compared with wild-type. It implies that the S-268019-b booster dose may have efficacy against recent epidemic variants, in line with previous literature [5].

Regarding T-cell responses, the S-268019-b booster dose elicited predominantly Th1-mediated immune reaction with minimal Th2 reaction in a cytokine profiling assay using peripheral blood mononuclear cells. There was no evidence of vaccine-mediated disease enhancement in our interim analysis, which concurs with the safety of Th1-biasing vaccines and contribution of Th2 pathology in vaccine-associated disease enhancement [16].

We acknowledge limitations of this study. The nonrandomized, open-label design may be prone to reporting bias [17]. The short follow-up duration for interim analysis and a relatively small sample size, especially of the elderly population, may preclude possibility of capturing rare AEs. Additionally, both elderly cohorts had a male sex predominance, which may influence the outcome. Finally, limited ethnic diversity, although intentional as a function of the study design, may limit generalizability of the results. Nonetheless, a heterologous booster dose of S-268019-b boosted immunity against SARS-CoV-2, with mostly low-grade reactogenicity in both adults and elderly Japanese participants with primary vaccinations with mRNA vaccines.

## Conclusion

S-268019-b vaccine containing SARS-CoV-2 spike protein and a squalene-based adjuvant was well tolerated when administered as a booster dose regardless of participants’ age. Neutralization antibodies, including those against Omicron variants, were induced after the booster dose in Japanese participants with prior vaccination with mRNA vaccines.

**Funding**

This work was supported by Shionogi & Co., Ltd., and Ministry of Health, Labour and Welfare (MHLW) under its supplementary budget for emergency maintenance associated with the vaccine production system. Preparation of clinical trial materials of S-268019-b was supported by 10.13039/100009619Japan Agency for Medical Research and Development (AMED) under Grant Number JP21nf010626.

## Declaration of Competing Interest

The authors declare the following financial interests/personal relationships which may be considered as potential competing interests: [Mari Ariyasu reports financial support was provided by Shionogi and Co Ltd. Mari Ariyasu reports financial support was provided by Ministry of Health, Labour and Welfare (MHLW). Mari Ariyasu reports equipment, drugs, or supplies was provided by Japan Agency for Medical Research and Development (AMED). Takuhiro Sonoyama, Akari Kamitani, Risa Y. Shibata, Naomi M. Seki, Shinya Omoto, Kenji Igarashi, and Mari Ariyasu are employees of Shionogi & Co., Ltd].

## Data Availability

Data will be made available on request.
