# Supplementary material for: Safety and immunogenicity of a booster dose of S-268019-b: Interim findings of a Phase 3, open-label clinical study in Japan
Source: Vaccine X. 2023 Sep 18;15:100390. doi: 10.1016/j.jvacx.2023.100390 (PMC10562875; doi:10.1016/j.jvacx.2023.100390)
Supplement: Supplementary data 1 [file mmc1.docx]

**Supplementary Table 1:** Potential AESIs

| **Neuroinflammatory disorders** | **Musculoskeletal disorders** | **Vasculitis** | **Others** |
| --- | --- | --- | --- |
| ●  Cranial nerve neuropathy, including paralysis and paresis (eg, Bell's palsy)  ●  Optic neuritis  ●  Multiple sclerosis  ●  Transverse myelitis  ●  Guillain-Barré syndrome, including Miller Fisher syndrome and other variants  ●  Acute disseminated encephalomyelitis, including site specific variants, eg, noninfectious encephalitis, encephalomyelitis, myelitis, myeloradiculoneuritis  ●  Myasthenia gravis, including Lambert-Eaton myasthenic syndrome  ●  Demyelinating peripheral neuropathies including:  –  Chronic inflammatory  demyelinating polyneuropathy  –  Multifocal motor neuropathy  –  Polyneuropathies associated with  monoclonal gammopathy  ●  Narcolepsy | ●  Systemic lupus erythematosus and associated conditions  ●  Systemic scleroderma  (systemic sclerosis), including:  –  Diffuse scleroderma  –  CREST syndrome  ●  Idiopathic inflammatory  myopathies, including:  –  Dermatomyositis  –  Polymyositis  ●  Anti-synthetase syndrome  ●  Rheumatoid arthritis and associated  conditions including:  –  Juvenile idiopathic  arthritis  –  Still's disease  ●  Polymyalgia rheumatica  ●  Spondyloarthropathies, including:  –  Ankylosing spondylitis  –  Reactive arthritis (Reiter’s  syndrome)  –  Undifferentiated spondyloarthritis  –  Psoriatic arthritis  –  Enteropathic arthritis  ● Relapsing polychondritis  ● Mixed connective tissue disorder  ● Gout | ●  Large vessels vasculitis including:  –  Giant cell arteritis (temporal arteritis)  –  Takayasu's arteritis  ●  Medium sized and/or small vessels vasculitis including:  –  Polyarteritis nodosa  –  Kawasaki's disease  –  Microscopic polyangiitis  –  Wegener's granulomatosis  (granulomatosis with polyangiitis)  –  Churg–Strauss syndrome (allergic granulomatous angiitis or eosinophilic granulomatosis with polyangiitis)  –  Buerger's disease (thromboangiitis  obliterans)  –  Necrotizing vasculitis (cutaneous or systemic)  –  ANCA-positive vasculitis (type unspecified)  –  Henoch-Schonlein purpura (IgA vasculitis)  –  Behcet's syndrome  –  Leukocytoclastic vasculitis | ●  Autoimmune glomerulonephritis including:  –  IgA nephropathy  –  Glomerulonephritis rapidly  progressive  –  Membranous glomerulonephritis  –  Membranoproliferative  glomerulonephritis  –  Mesangioproliferative  glomerulonephritis  –  Tubulointerstitial nephritis and  uveitis syndrome  ●  Ocular autoimmune diseases including:  –  Autoimmune uveitis  –  Autoimmune retinitis  ●  Autoimmune myocarditis  ●  Sarcoidosis  ●  Stevens-Johnson syndrome  ●  Sjögren's syndrome  ●  Alopecia areata  ●  Idiopathic pulmonary fibrosis  ●  Goodpasture syndrome  ●  Raynaud's phenomenon |
| **Skin disorders** | **Blood disorders** | **Gastrointestinal disorders** | **Endocrine disorders** |
| ●  Psoriasis  ●  Vitiligo  ●  Erythema nodosum  ●  Autoimmune bullous skin diseases  (including pemphigus, pemphigoid, and dermatitis herpetiformis)  ●  Lichen planus  ●  Sweet's syndrome  ●  Localized scleroderma (morphea) | ●  Autoimmune hemolytic anemia  ●  Autoimmune thrombocytopenia  ●  Antiphospholipid syndrome  ●  Pernicious anemia  ●  Autoimmune aplastic anemia  ●  Autoimmune neutropenia  ●  Autoimmune pancytopenia | ●  Inflammatory bowel disease, including:  –  Crohn's disease  –  Ulcerative colitis  –  Microscopic colitis  –  Ulcerative proctitis  ●  Celiac disease  ●  Autoimmune pancreatitis | ●  Autoimmune thyroiditis (Hashimoto thyroiditis)  ●  Grave's or Basedow's disease  ●  Diabetes mellitus type 1  ●  Addison's disease  ●  Polyglandular autoimmune syndrome  ●  Autoimmune hypophysitis |
| **Liver disorders** |  |  |  |
| ●  Autoimmune hepatitis  ●  Primary biliary cirrhosis  ●  Primary sclerosing cholangitis  ●  Autoimmune cholangitis |  |  |  |

AESI, adverse events of special interest; ANCA, Antineutrophil cytoplasmic antibody; IgA, immunoglobulin A.
